# Title of report: second primary ovarian carcinomas after breast cancer diagnosis- an analysis of a single cancer centre in China

**DOI:** 10.3389/fonc.2025.1553366

**Published:** 2025-06-03

**Authors:** Hong Liu, Min Luo, Chunrong Peng, Xinghan Cheng, Gupo Luo, Dengfeng Wang, Guonan Zhang

**Affiliations:** ^1^ Gynecologic Oncology Center, Sichuan Clinical Research Center for Cancer, Sichuan Cancer Hospital & Institute, Sichuan Cancer Center, Affiliated Cancer Hospital of University of Electronic Science and Technology of China, Chengdu, China; ^2^ Department of Oncology, The Sixth People’s Hospital of Chengdu, Chengdu, China

**Keywords:** double primary malignancy, breast cancer, ovarian cancer, interval between diagnoses, survival outcome

## Abstract

**Objective:**

We aimed to evaluate the clinicopathological characteristics and survival outcomes of patients with second primary ovarian carcinomas after a breast cancer diagnosis.

**Materials and methods:**

We reviewed the medical reports of 23 patients at Sichuan Cancer Hospital between May 2002 and October 2021. We analyzed demographic and clinicopathologic characteristics, the time interval between diagnoses, and survival time. Kaplan-Meier and Cox proportional hazards regression tests were used to determine survival outcomes.

**Results:**

The median age of patients at the time of diagnosis of breast cancer (BC) and ovarian cancer (OC) was 46 and 49 years, respectively. Among them were 6 cases of synchronous OC and 17 instances of metachronous OC. The average interval between diagnoses of the two cancers was 62.48 months. The median OS after the second primary OC diagnosis was 38 months. According to Kaplan-Meier’s analysis, the advanced stage at presentation of BC (p=0.023) resulted in a significantly shorter interval between BC and OC diagnosis. On univariate Cox regression analysis, only BC Scarff-Bloom-Richardson (SBR) 3 Grade resulted in a considerably worse PFS (HR 0.187, p=0.048) and OS (HR 0.190, p=0.048), respectively.

**Conclusion:**

We should strengthen the follow-up management of breast cancer patients.The later the stage of breast cancer, the shorter the time interval of diagnosis of OC was. Early control of ovarian tumors and active comprehensive treatment for synchronous and metachronous breast and ovarian cancer can achieve good results.

## Highlights

- We should strengthen the follow-up management of breast cancer patients.
- The later the stage of breast cancer, the shorter the time interval of diagnosis of OC was.
- Early control of ovarian tumors and management throughout the treatment of BC and OC can yield good result.

## 1 Introduction

Multiple primary cancer (MPC) refers to the simultaneous or sequential occurrence of two or more primary cancers in the same patient’s single or multiple organs, with the same or different tissue types but no subordinate relationship between the tumors. Synchronous tumors are defined as the diagnosis of both primary tumors at the same time or second primary cancers detected within six months of primary tumor detection, while metachronous tumors have been defined as the diagnosis of both primary tumors more than six months apart.

In recent years, more and more patients with breast cancer have ovarian cancer many years later. Still, the diagnosis and treatment of some patients are often delayed due to a lack of attention. Lee et al. reported that the incidence of recurrent breast cancer second tumors in breast cancer was 2.02%, of which 0.34% were patients with ovarian cancer (1). Young women with a history of breast cancer were at high risk for ovarian cancer even 20 years after diagnosis (2). It is known that both breast and ovary are target organs of the sex hormone regulation axis, and they also have some common regulatory genes (3, 4), which may be the disease basis of breast cancer combined with ovarian cancer. Hereditary breast cancer ovarian cancer syndrome (HBOCS) is the most common underlying cause of hereditary breast cancer, with an incidence of 2%~7% in all breast cancer (5). BC and OC presenting as a synchronous or metachronous tumour often pose a diagnostic dilemma.There are many similarities between the primary ovarian cancer and the ovarian metastases from breast cancer but the treatment methods and prognosis between the two groups are different. So we should be focused on the differential diagnosis of metastatic disease and primary ovarian neoplasms.

There is little known regarding the time intervals between initial BC and OC diagnosis, as well as the prognosis of people with both cancers. So our study looked at the clinicopathological characteristics, time intervals between these two primary tumors, and survival outcomes of patients with second primary ovarian carcinomas after a breast cancer diagnosis.

## 2 Materials and methods

### 2.1 Patients

We reviewed medical reports of patients with second primary ovarian carcinomas after breast cancer diagnosis between January 2002 and December 2021 at Sichuan Cancer Hospital. The information includes demographic data, tumor stage (BC staging according to the 8th edition American Joint Committee on Cancer staging system while OC staging according to the 2018 International Federation of Gynecology and Obstetrics staging system), histological type, tumor grade, hormonal/HER2 receptors status (BC), surgical and medical treatment, the time intervals between double primary breast and ovarian cancers and disease-related outcomes, et al. were collected. The Institutional Review Board of Sichuan Cancer Hospital approved this study. The requirement for informed consent was waived because we analyzed de-identified data secondarily.

### 2.2 Pathology

Synchronous and Metachronous Breast and Ovarian Cancer was confirmed using a panel of IHC markers, PAX8, WT1, P53, CA125, and P16 for OC, and estrogen receptor (ER), progesterone receptor (PR), HER2, mammaglobin, GCDFP-15, GATA3 for BC. Additional markers such as CK7, CK20, and Vimentin were performed to rule out metastatic disease from the gastrointestinal tract and when required. These markers were used to approximately classify patients into four breast cancer subtypes: luminal A (ER + or PR +, and HER2 -); luminal B (ER + or PR +, and HER2 +); HER2 enhanced (ER -, PR- and HER2 +); and triple negative (ER -, PR - and HER2-).

### 2.3 Statistical analysis

Statistical analysis was conducted using SPSS statistical software version 27 (IBM Corp.). The association between variables was evaluated using the χ2 or t-test, as appropriate. Overall survival (OS) was calculated as the interval between each cancer diagnosis and death or last follow-up. We also evaluated the progression-free survival (PFS) after the OC diagnosis, calculated from the time of OC diagnosis to progression or last follow-up. Survival curves were estimated using the Kaplan–Meier model and the hazard ratio (HR) and 95% confidence interval (95% CI) were calculated with the Cox regression model. Level of significance was set at 0.05. The data cut-off for the survival events was March 2024.

## 3 Results

### 3.1 Clinico-pathological characteristics

A total of 23 patients were included in the study, and the median age at BC or OC diagnosis was 46 years (32-62 years)and 49 years (40-71 years), respectively. Among them were 6 synchronous OC and 17 cases of metachronous OC. Only one patient has a family history of tumors; her mother suffers from rectal cancer, and her brother suffers from lung cancer. Approximately 56.5% of OC cases occurred during the first 60 months after the BC diagnosis. The most common histological subtype of OC was high-grade serous carcinoma (19/24, 82.6%), other 4 patients was endometrioid adenocarcinoma. At the time of diagnosis of OC, the median serum CA-125 levels were 448.7 U/mL (16.4-3578 U/mL) (**Table 1**).

**Table 1:** Clinicopathological characteristics of patients of BC diagnosis and OC diagnosis.

Patient no	Age at BC diagnosis (years)	BC stage	Surgical approach	Histological Type of BC	SBR Grade	Immunophenotype(BC)	Survival time after diagnosis of BC (Months)	Age at OC diagnosis (years)	OC stage	Interval between diagnosis of BC and OC (months)	Treatment	CA125(U/ml)	BRCA mutation status	Parpi maintenance treatment	PFS after diagnosis of OC (months)	Survival outcome	Survival time after diagnosis of OC (Months)
1	32	IIA	1	1	1	3	178	40	IIIc	100	1	3578	BRCA+	Olaparib second-line	15	1	82
2	56	IIB	2	1	2	4	55	58	IIa	13	1	602	BRCA+	Olaparib	27	1	40
3	51	IA	2	2(apocrine carcinoma)	2	3	51	51	IIIc	13	1	2463	BRCA+	Olaparib second-line	26	1	38
4	45	IA	1	1	2	1	47	45	IIIc	0	1	1305	Unknown	NA	47	3	47
5	58	IA	2	1	1	1	226	67	IIIc	204	2	512	BRCA+	Olaparib	18	1	18
6	46	IA	2	1	2	1	75	46	IV	0	2	274.4	Unknown	NA	34	3	75
7	41	IIIA	2	1	2	4	82	45	IV	60	2	512.4	Unknown	NA	7	2	23
8	46	IIB	2	1	1	2	130	55	IC	116	1	16.4	Unknown	NA	12	2	14
9	48	IIIC	2	1	2	2	14	48	IIIC	0	1	448.7	Unknown	NA	3	2	14
10	35	IIIA	1	1	2	4	80	40	IIIc	56	2	1092	Unknown	Nilapali first-line maintenance therapy	10	1	24
11	45	IIIA	2	2(cribriform carcinoma)	1	2	52	45	IIIc	0	1	409	Unknown	NA	32	2	52
12	46	IIA	1	2(cephaloma)	1	3	111	53	IIa	85	1	291.7	Unknown	NA	8	3	27
13	43	IIA	2	1	2	3	84	43	IIIa	0	1	455.8	Unknown	NA	14	3	84
14	47	IIB	2	2(invasive micropapillary carcinoma)	1	2	178	57	IIIc	144	2	321	Unknown	NA	13	2	49
15	26	IIA	2	1	1	3	259	46	IIIc	235	2	158.3	Unknown	NA	15	2	24
16	54	IIA	2	1	2	2	130	59	IV	74	2	31.6	Unknown	NA	41	1	60
17	41	IIA	2	1	2	2	144	49	IIIc	89	2	3413	Unknown	NA	5	3	55
18	44	IIB	2	1	2	2	112	47	IIb	38	1	104.5	BRCA+	Olaparib	27	1	74
19	42	IIIA	1	1	2	2	150	53	IV	133	3	960.5	Unknown	NA	13	1	15
20	62	IIA	2	1	1	3	72	67	IV	58	3	470	Unknown	NA	7	2	14
21	50	IIA	1	1	2	1	29	51	IIIa	9	3	105	Unknown	Nilapali	20	1	20
22	49	IA	2	1	2	3	46	48	IIIc	0	1	245	Unknown	NA	22	1	22
23	46	IIB	1	1	2	2	138	71	Ia	10	1	56	Unknown	NA	83	2	128

Surgical approach:1=breast conservation surgery(BCS), 2=modifed radical mastectomy(MRM).

Histological type of BC: 1=Invasive Ductal Carcinoma(IDC).

2=Special infiltrative breast carcinoma(SIBC).

Scarff-Bloom-Richardson grade(SBR Grade): 1=1-2 Grade, 2=3 Grade.

Immunophenotype(BC): 1=Luminal A, 2=Luminal B, 3=HER2 enhanced, 4=Triple negative.

Treatment:1=PDS+chemotherapy, 2=IDS+chemotherapy, 3=Chemotherapy.

Survival outcome: 1=alive, 2=dead, 3=unknown.

NA, not applicable.

All patients were treated following discussion in a multidisciplinary team. Surgery for BC comprised modified radical mastectomy (MRM) and breast conservation surgery (BCS), followed by chemo-endocrine therapy and radiotherapy as per institutional protocols depending on the stage and immunophenotype of breast cancer. For patients with OC, 3 patients received chemotherapy alone due to extensive lesions or refusal of surgery. Among 6 cases of synchronous OC who were all diagnosed at the same time with BC, five patients underwent PDS and one patient underwent IDS; considering that the prognosis of ovarian cancer is worse than that of breast cancer, all patients firstly underwent surgery for ovarian cancer and adjuvant chemotherapy then obtained breast cancer surgery (**Table 2**).

**Table 2:** Univariate analysis of interval time between diagnosis of BC and OC and Median PFS and OS of 23 patients.

Variables	Case (N)	Median interval time between diagnosis of BC and OC (months)	P	Median PFS after diagnosis of OC (months)	P	Median Survival time after diagnosis of OC (months)	P
Age at BC diagnosis(years)			0.506		0.540		0.781
≤50	18	144		32		128	
>50	5	NR		NR		NR	
Menopausal status at BC diagnosis			0.506		0.540		0.781
No	18	144		32		128	
Yes	5	NR		NR		NR	
BC stage			0.023		0.084		0.111
I-IIA	13	235		NR		NR	
IIB-IV	10	116		32		52	
BC SBR Grade			0.882		0.026		0.024
1-2	8	144		15		49	
3	15	NR		83		128	
BC Immunophenotype			0.352		0.449		0.710
Luminal A	4	–		–		–	
Luminal B	9	–		–		–	
HER2 enhanced	7	–		–		–	
Triple negative	3	–		–		–	
Ki 67 index status			0.525		0.220		0.152
<20%	8	116		32		52	
≥20%	15	144		NR		NR	
BC histological type			0.603		0.472		0.550
IDC	19	235		83		128	
SIBC	4	144		32		49	
BC surgical approach			0.524		0.063		0.083
BCS	7	NR		83		128	
MRM	16	116		32		52	
Age at OC diagnosis(years)			–		0.989		0.761
<60	20	–		NR		NR	
≥60	3	–		83		128	
OC Stage			–		0.615		0.560
I-II	5	–		83		128	
III-IV	18	–		NR		NR	
OC histological type			–		0.456		0.631
Serous adenocarcinoma	19	–		NR		NR	
Endometrioid adenocarcinoma	4	–		13		49	
Histological grade of OC			–		0.336		0.268
High grade	21	–		NR		NR	
Low grade	2	–		83		128	
Time Intervals between BC and OC diagnosis (months)			–		0.155		0.371
<24	10	–		83		128	
≥24	13	–		15		NR	
OC treatment			–		0.770		0.499
PDS+chemotherapy	12	–		83		128	
IDS+chemotherapy	8	–		NR		NR	
Chemotherapy	3	–		NR		NR	
OC location			–		0.593		0.978
Unilateral	8	–		32		52	
Bilateral	15	–		NR		NR	
CA125 at OC diagnosis(u/ml)			–		0.092		0.069
<600	16	–		–		–	
≥600	7	–		–		–	
BRCA mutation status			–		0.120		0.135
BRCA(+)	5	–		–		–	
Unknown	18	–		–		–	
Parpi maintenance treatment			–		0.062		0.083
No	16	–		–		–	
Yes	7	–		–		–	

NR, Not Reached.

### 3.2 Long-term outcome

Five patients (21.7%) was lost to follow-up, 8 patients (34.8%) died due to progression of OC. The average interval between BC and OC diagnosis was 62.48 months (**Table 2**). The median PFS and OS after OC diagnosis was 15 months and 38 months, respectively.

**Figure 1:**
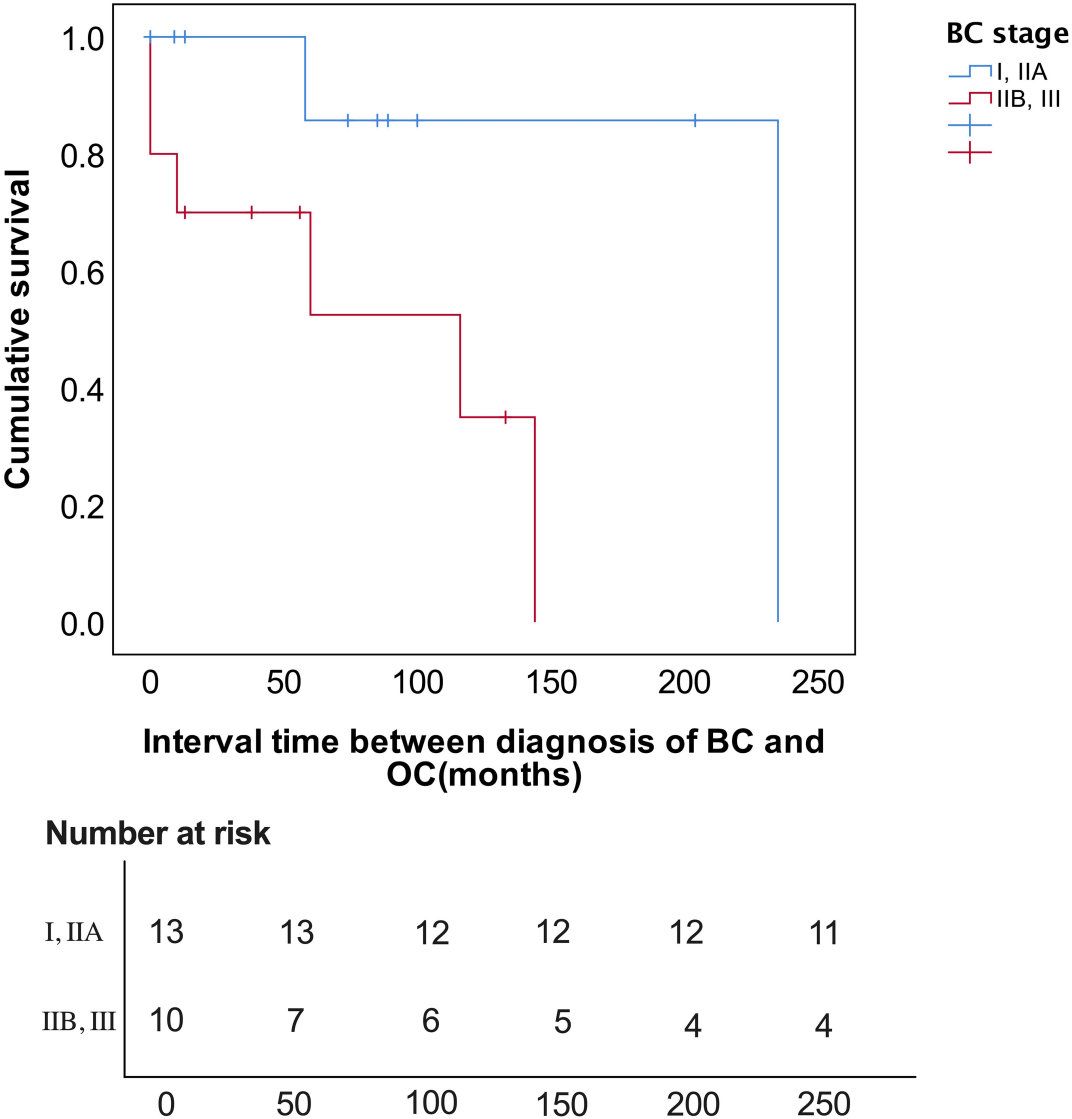
Kaplan-Meier survival curve for breast cancer stage.

On Kaplan-Meier analysis, the advanced stage at presentation of BC (p=0.023) resulted in a significantly shorter interval time between BC and OC diagnosis (**Figure 1**). On the univariate Cox regression analysis, it was also found that the later the BC stage was, the shorter interval time between the diagnosis of BC and OC was (HR 8.047, p = 0.055), although it was not statistically significant (**Table 3**). On univariate Cox regression analysis, only BC SBR Grade 3 resulted in a significantly worse PFS (HR 0.187, p=0.048) and OS (HR 0.190, p=0.048), respectively (**Table 4**).

**Table 3:** Cox regression univariate of influence factors of interval time between BC and OC diagnosis.

Variables		Univariable analysis	
		HR (95% CI)	*p*-value
Age at BC diagnosis(years)	≤50	1	
	>50	0.495(0.059-4.150)	0.517
Menopausal status at BC diagnosis	No	1	
	Yes	0.495(0.059-4.150)	0.517
BC stage	I-IIA	1	
	IIB-IV	8.047(0.957-67.689)	0.055
BC SBR Grade	1-2	1	
	3	1.136(0.208-6.205)	0.883
BC Immunophenotype	Luminal A	1	
	Luminal B	50465.481(0.000-1.540E+139)	0.945
	HER2 enhanced	14403.279(0.000-4.417E+138)	0.952
	Triple negative	51968.315(0.000-1.596E+139)	0.945
Ki 67 index status	<20%	1	
	≥20%	0.612(0.132-2.846)	0.531
BC histological type	IDC	1	
	SIBC	1.542(0.295-8.077)	0.608
BC surgical approach	BCS	1	
	MRM	1.975(0.230-16.993)	0.535

**Table 4:** Cox regression univariate analyses of influence factors of PFS and OS after OC diagnosis.

Variables		Univariable analysis (PFS)		Univariable analysis (OS)	
		HR (95% CI)	*p*-value	HR (95% CI)	*p*-value
Age at BC diagnosis(years)	≤50	1		1	
	>50	0.524(0.063-4.366)	0.550	0.745(0.089-6.255)	0.787
Menopausal status at BC diagnosis	No	1		1	
	Yes	0.524(0.063-4.366)	0.550	0.745(0.089-6.255)	0.787
BC stage	I-IIA	1		1	
	IIB-IV	3.820(0.737-19.805)	0.110	3.419(0.662-17.653)	0.142
BC SBR Grade	1-2	1		1	
	3	0.187(0.036-0.982)	0.048	0.190(0.037-0.986)	0.048
BC Immunophenotype	Luminal A	1		1	
	Luminal B	109544.112(0.000-3.353E+180)	0.955	30595.828(0.000-5.281E+136)	0.947
	HER2 enhanced	83290.559(0.000-2.554E+180)	0.956	24234.811(0.000-4.189E+136)	0.948
	Triple negative	130219.311(0.000-4.003E+180)	0.954	34317.964(0.000-5.952E+136)	0.946
Ki 67 index status	<20%	1		1	
	≥20%	0.406(0.091-1.820)	0.239	0.356(0.079-1.603)	0.178
BC histological type	IDC	1		1	
	SIBC	1.802(0.349-9.301)	0.482	1.633(0.312-8.541)	0.561
BC surgical approach	BCS	1		1	
	MRM	38.134(0.050-28909.831)	0.282	34.957(0.035-34822.082)	0.313
Age at OC diagnosis(years)	<60	1		1	
	≥60	0.985(0.118-8.229)	0.989	1.380(0.164-11.591)	0.767
OC Stage	I-II	1		1	
	III-IV	1.704(0.205-14.175)	0.622	1.839(0.221-15.300)	0.573
OC histological type	Serous adenocarcinoma	1		1	
	Endometrioid adenocarcinoma	1.852(0.353-9.718)	0.466	1.487(0.284-7.801)	0.639
Histological grade of OC	High grade	1		1	
	Low grade	0.040(0.000-1151.335)	0.540	0.037(0.000-425.315)	0.490
Time Intervals between BC and OC diagnosis (months)	<24	1		1	
	≥24	3.222(0.588-17.670)	0.178	2.052(0.398-10.592)	0.391
OC treatment	PDS+chemotherapy	1		1	
	IDS+chemotherapy	1.692(0.341-8.405)	0.520	1.443(0.291-7.165)	0.654
	Chemotherapy	1.801(0.181-17.891)	0.616	3.924(0.314-49.058)	0.289
OC location	Unilateral	1		1	
	Bilateral	1.553(0.301-8.012)	0.599	1.023(0.196-5.329)	0.978
CA125 at OC diagnosis(u/ml)	<600	1		1	
	≥600	0.029(0.000-30.397)	0.318	0.027(0.000-23.585)	0.296
BRCA mutation status	BRCA(+)	1		1	
	Unknown	34.894(0.018-67529.082)	0.358	30.300(0.017-53706.882)	0.369
Parpi maintenance treatment	No	1		1	
	Yes	0.024(0.000-25.104)	0.292	0.029(0.000-28.496)	0.313

## 4 Discussion

In this article, the average age of BC was 45.78 years and 73.9% of them were under 50 years old. 78.3% patients were in premenopausal. Tong et al. (6) reported that MPC patients onset 10-20 years earlier than primary epithelial ovarian cancer (average age 56-60 years) and are more common in premenopausal women. Berkowitz et al. (2) found that for women diagnosed with BC at an age younger than 50, the relative risk (RR) of OC diagnosed within two months to 5 years was 2.5 [95% confidence interval (CI): 2.1 -2.9]. Thus, it can be seen that the risk of postoperative OC in young patients with BC is significantly higher, which is similar to the results of this study.

Patients with double primary BC and OC are sporadic. Suppose women have a history of unmarried and childless, family inheritance, triple-negative breast cancer, postoperative oral selective estrogen antagonist, and BRCA1/BRCA2 gene mutation. In that case, they should be particularly alert to the possibility of the occurrence of OC after breast cancer surgery. The most important risk factors are the family history of the tumor (especially the medical history of first-degree relatives) and BRCA1/2 gene mutations (7). The estimated risk of developing ovarian cancer is increased approximately 2-fold for patients with a history of breast cancer (8). Metcalfe et al. reported a 10-year actuarial risk of developing EOC after BC of 12.7% for BRCA1 and 6.8% for BRCA2 mutation carriers (p=0.03) (9). In our study, due to the lack of widespread testing technology and high testing costs in earlier years, the vast majority of patients had not undergone BRCA gene mutation testing. Five patients had BRCA germline mutation. Thus, it can be seen that BRCA1/2 gene screening is essential for the prevention and early detection of hereditary breast cancer/ovarian cancer. By accurately stratifying patients’ risk and guiding targeted screening and preventative interventions, development of a novel prediction model for carriage of BRCA1/2 pathogenic variant in patients with breast cancer will contribute to improved management and outcomes of HBOC (10). In this article, only one patient has a family history of tumors, which suggesting that double primary breast and ovarian tumors can also appear in those without prior relevant clinical or family histories and other common etiological factors such as hormonal and reproductive aspects and mutation of different genes involved in tumor suppression may also induce cancer to occur (11–13).Other relevant genes are related to HBOC syndrome diagnosis, prognosis, and treatment, including TP53, PALB2, CHEK2, ATM, etc. Multi-gene testing implementation improves the detection of often overlooked genes related to HBOC pathogenesis and treatment (14). The majority of cases of breast cancer were initial (IA - IIA), luminals and treated with radical mastectomy in our study,Generally, this type of breast cancer has a good prognosis, but the simultaneous occurrence of ovarian cancer in this type of patients indirectly proves that genetic factors such as BRCA mutations may lead to secondary ovarian cancer rather than metastasis.In our study, 5 patients had BRCA mutations, and 18 patients had unknown BRCA detection status. The study showed that the mutation status of BRCA did not affect the survival of the patients.As several reports suggested (15–17), the initial survival advantage among EOC patients with BRCA mutations may reflect a higher initial sensitivity of BRCA carriers to chemotherapy and short-term survival but this response does not predict long-term survival. The strongest predictor of long-term survival is the status of no residual disease at resection. Though BRCA mutations appear to be associated with improved OS and PFS in patients with ovarian cancer, there has no difference in the surgical resection rate between participants in the two groups (18). Another study also proves that BRCA-status is not a prognostic factor in early ovarian cancer regarding PFS (19). It should emphasize the major impact of achieving complete surgery on the prognosis of HRD EOCs with or without BRCA1/2 mutation, whether in primary surgery or interval surgery, despite a better sensitivity to chemotherapy and maintenance treatments with PARP inhibitors (20).

In our study, the average interval between BC and OC diagnosis was 62.48 months, and approximately 43.5% of second primary OC cases occurred after the first 60 months of the BC diagnosis, suggesting that breast cancer patients who have achieved long-term survival should also be alert to the occurrence of ovarian cancer. Metcalfe et al. (9) found a mean time of 8.1 years (range 0.1–25.5 years) from BC to OC. In this article, the Kaplan-Meier approach revealed that the later the stage of breast cancer, the shorter the time interval of diagnosis of OC was. It reminds doctor to strengthen the follow-up management of breast cancer patients, especially the screening management of high-risk patients with ovarian cancer. During the follow-up of breast cancer, attention should be paid to gynecological examination and gynecological ultrasound examination. If the patient is taking tamoxifen and the uterus and ovary are not removed surgically, they should be examined every 3-6 months. If patients with BRCA mutations are recommended to undergo prophylactic bilateral adnexectomy to reduce the risk of ovarian cancer. One meta-analysis revealed that prophylactic interventions significantly reduced cancer risk and mortality. Risk-reducing surgeries (RRS) were more effective than chemoprevention, with RRS notably reducing cancer risk by 56% compared to 39% for chemoprevention (21). Joshi S suggested timing prophylactic oophorectomy 5–6 years after diagnosis of first BC based on the median time interval before development of second cancer to be 77 months after diagnosis of OC (22). Our study found that approximately 56.5% of OC cases occurred during the first 60 months after the BC diagnosis, therefore, prophylactic oophorectomy should be performed more earliar.

In our study, most of the patients were bilateral tumors (65.2%), ovarian tumors’ size was >5cm (73.9%), the pathological types of ovarian cancer were mostly serous papillary carcinoma and endometrioid carcinoma, and the ovarian tumor was located in the epithelium. The main emphasis should be to differentiate BC from primary OC and vice versa for subsequent best management of these patients to decide curative or palliative approach. The clinicopathological characteristics of breast cancer with ovarian metastasis are as follows: most of them have no obvious symptoms, and a few of them have symptoms, such as abdominal distention, ascites, abnormal vaginal bleeding, etc (14).Breast metastases to the ovary most frequently are bilateral solid masses at ultrasonographic (US) with small tumor size, multiple nodules and involvement of the surface (17, 23–26), while patients with primary OC had predominantly cystic images at US (27). Previous tumor history is an important factor to assist in the differential diagnosis of primary or metastatic ovarian cancer. It is reported that patients with BC are 3-7 times more likely to develop primary ovarian cancer (POC) than ovarian metastases (OM) (28). A single imaging or CA125 examination is not meaningful in distinguishing between primary and metastatic ovarian tumors. Many ovarian metastatic adenocarcinoma and primary ovarian cancer have extremely similar pathological morphology and clinical pathological types, requiring careful pathological identification. Immunohistochemistry has a pivotal role in differentiating primary and secondary ovarian adenocarcinoma. In our study, ovarian tumor cell immunohistochemistry showed positive expression of P53, WT-1, PAX8, Ck7, P16, ER, and CA125, while GCDFP-15, mammaglobin, GATA3, CK20, Vimentin expression were negative. WT1 and PAX8 appear to have utility in differentiating primary OC from metastatic BC due to their higher sensitivity and low potential for aberrant expression (29). GATA3 is a very specific marker for breast cancer (and urothelial carcinomas) (30). Wick et al. reported that the overall sensitivity of GCDFP 15 in the diagnosis of breast cancer is 74%, the specificity is 95%, and the negative predictive value is 95% (31). Monteagudo et al.found that GCDFP-15 was positive in 71% of breast cancer metastatic to the ovary, while none of the primary ovarian cancer was positive (32). Mammaglobin, GCDFP-15, CK7, and GATA-3 are the most commonly used markers to confirm breast origin. Because none of these markers is completely specific and/or sensitive, ovarian markers should be included to obtain the differential diagnosis (33).

In our study, there were 6 cases of synchronous OC. Among them, 5 patients underwent PDS and one patient underwent IDS. All of them firstly underwent surgery for ovarian cancer and adjuvant chemotherapy then obtained breast cancer surgery. Primary BC and OC in the same patient must be treated following the best evidence available for each tumor, considering disease stage, pathology findings and molecular characteristics. Currently, there is no standard treatment of patients with synchronous OC and BC. Neoadjuvant chemotherapy was effective in both breast and ovarian cancer. We used platinum drugs, taxanes, and anthracycline agents, which were active in both diseases. In the context of advanced disease, treatment may be tailored according to prognosis, patient´s performance status and preferences. According to research reports, early control of ovarian tumors with poorer prognosis can avoid distant metastasis and widespread implantation metastasis as early as possible (34–36). In our study, all of patients were died of OC progression. Studies reported that OS survival was dominated by the stage of disease (37, 38) and the most virulent of the synchronous tumors defined mortality rates, while the mortality rate of individuals with metachronous tumors was determined by second malignancies after the first neoplasm was cured (39).

As we known,PARP inhibitors (olaparib, niraparib) are effective targeted therapies in BRCAm ovarian cancer in first-line treatment as well as in maintenance therapy after platinum based chemotherapy for ovarian cancer recurrence (40–43). In our study,the vast majority of patients did not receive maintenance treatment with PARPi due to economic reasons and the fact that PARPi was only approved for ovarian cancer in China after 2018. Only 6 patients received PARP inhibitor maintenance treatment, among them, four patients receiving first-line maintenance therapy, including two BRCA mutation patients receiving olaparib maintenance therapy, two patients with unknown genetic testing status receiving nirapali maintenance therapy, and two second-line maintenance therapy patients with BRCA mutations receiving olaparib maintenance therapy. PARP inhibitors reduced the risk of disease progression and death (HR=0.024) and (HR= 0.029), respectively, although it was not statistically significant, which may be related to only a few patients in our study had received PARPi maintenance treatment.

To the best of our knowledge, this is one of the few study to investigate the time intervals and prognosis of individuals with both primary BC and OC. But it also has several shortcomings. Firstly, It was retrospective study and the sample size was relatively small which limits the generalizability of the conclusions. Secondly, there were a limited number of patients who underwent BRCA1/2 gene testing and maintenance therapy. Thirdly, the analysis did not include the assessment of treatment after OC recurrence. Future studies with larger sample sizes and more rigorous research designs are necessary to further elucidate the prognostic value of variables such as age, disease characteristics, mutation type, targeted therapy, intervention, surveillance and prophylactic surgery and to identify the appropriate management strategies for this specific population.

In conclusion, patients with breast cancer may have ovarian cancer later,it reminds doctor to strengthen the follow-up management of breast cancer patients, especially the screening management of high-risk patients with ovarian cancer. Early control of ovarian tumors and active comprehensive treatment for synchronous and metachronous breast and ovarian cancer can achieve good results.

## Data Availability

The raw data supporting the conclusions of this article will be made available by the authors, without undue reservation.
